# Health literacy and prevention behavior for preventing premature pregnancy among ethnic students in Northern Thailand

**DOI:** 10.3389/fpubh.2025.1718974

**Published:** 2026-01-21

**Authors:** Soontaree Suratana, Paitoon Yodkard, Anusorn Udplong, Thanatchaporn Mulikabut, Ratipark Tamornpark

**Affiliations:** 1 School of Health Science, Mae Fah Luang University, Chiang Rai, Thailand; 2 Area-Based Research and Innovation in Cross-Border Healthcare Group, Mae Fah Luang University, Chiang Rai, Thailand; 3 Faculty of Engineering, Chiang Rai College, Chiang Rai, Thailand; 4 Center of Excellent for Hill-Tribe Health Research, Mae Fah Luang University, Chiang Rai, Thailand

**Keywords:** ethnic adolescents, health literacy, prevention behavior, reproductive health, teenage pregnancy, Thailand

## Abstract

**Background:**

Premature pregnancy among adolescents remains a critical public health challenge, particularly among ethnic populations in low-resource settings. Health literacy has been identified as a key determinant of reproductive decision-making and preventive behaviors. This study aims to assess the levels of health literacy and examine their association with pregnancy prevention behavior among ethnic students in northern Thailand.

**Methods:**

A cross-sectional study was conducted with 128 in-school ethnic adolescent girls, using validated instruments to measure health literacy across three domains: basic, interactive, and critical health literacy, and pregnancy prevention behavior. Descriptive statistics, Pearson's correlation, and multiple linear regression were used to analyze the data.

**Results:**

The participants exhibited moderate overall health literacy, with high scores in decision-making skills but relatively low scores in basic and interactive literacy. Pearson's correlation analysis revealed that critical literacy was positively associated with prevention behavior (*r* = 0.336, *p* < 0.001), while interactive literacy showed a negative correlation (*r* = −0.247, *p* < 0.001). Multiple regression analysis further confirmed these associations. Critical literacy significantly predicted pregnancy prevention behavior (β = 0.438, *p* < 0.001), whereas interactive literacy had a significant adverse effect (β = −0.568, *p* < 0.001). The model explained 17.8% of the variance in behavior (*R*^2^ = 0.178).

**Conclusion:**

The findings underscore the importance of critical health literacy in fostering preventive behaviors among ethnic adolescents. Tailored interventions should emphasize evaluative and judgmental skills while addressing culturally rooted communication challenges. These insights may inform inclusive reproductive health policies in ethnic and underserved communities.

## 1 Introduction

Teenage pregnancy remains a significant global public health challenge, with far-reaching social, economic, and health consequences. Each year, an estimated 12.8 million girls aged 15–19 give birth, most of whom reside in low and middle-income countries (LMICs) (1–3). Adolescents defined by the World Health Organization as individuals aged 10–19 years (4). Adolescent mothers face elevated risks of obstetric complications, mental health disorders, and school dropout, while their children are more likely to experience poor physical and developmental health outcomes such as low birthweight, prematurity, and early childhood malnutrition, leading to increased vulnerability to intergenerational poverty (5–7). Recognizing the scale of this challenge, the United Nations has made the prevention of adolescent pregnancy a core component of Sustainable Development Goal (SDG) 5.6, which advocates for universal access to sexual and reproductive health and rights (8). Despite growing international commitments, national-level progress in countries such as Thailand has been uneven. Although Thailand has introduced policies such as the Prevention and Solution of the Adolescent Pregnancy Problem Act (2016) and national strategies to reduce teenage births, disparities persist across regions and among specific populations (9, 10). Among the most vulnerable are ethnic minority adolescents living in remote highland areas of northern Thailand. These groups—such as the Akha, Lahu, Hmong, and Karen—often face multidimensional marginalization, including limited access to formal education, healthcare, and culturally appropriate reproductive health services (11–14).

Emerging evidence indicates that ethnic minority adolescents experience overlapping and compounding reproductive health disadvantages shaped by multi-level racism, gendered power dynamics, and structural marginalization. These intersecting factors limit their autonomy, narrow their reproductive choices, and systematically constrain their access to equitable health information and services (15). Traditional beliefs often influence reproductive decisions more than formal health education. For instance, early marriage is often culturally endorsed, and discussions about contraception and sexual health may be taboo within families or communities (16). Moreover, language barriers and limited exposure to Thai-language health media further exacerbate their exclusion from mainstream health systems. Such conditions restrict their ability to access accurate information, negotiate safe behaviors, and make autonomous reproductive choices.

Amid these structural and cultural challenges, health literacy has emerged as a key factor influencing reproductive health outcomes. Defined by Nutbeam as comprising functional, interactive, and critical competencies, health literacy empowers individuals to obtain, process, and apply health information effectively (17, 18). However, its role among ethnic adolescents whose cultural logic, educational experiences, and social norms differ considerably from those of the majority population has not been adequately explored (19). Global evidence suggests that improving health literacy can lead to better sexual and reproductive health outcomes, such as increased contraceptive uptake, delayed sexual initiation, and reduced unintended pregnancy (20), but interventions often fail when they neglect local belief systems and lived experiences (21).

In the context of northern Thailand, the problem is particularly pressing. Chiang Rai Province, home to over 200,000 ethnic residents, has one of the highest adolescent pregnancy rates in the country. For many of these adolescents, reproductive decision-making occurs within a web of community expectations, family pressures, and limited-service accessibility (22, 23). Mainstream interventions, often designed without the input of ethnic communities, may be ineffective or even counterproductive if perceived as culturally alien.

This study aims to fill a critical gap by examining the relationship between health literacy and pregnancy prevention behaviors among school-aged ethnic adolescent girls in northern Thailand. Grounded in Nutbeam's multidimensional framework, the study examines how various dimensions of literacy, such as access to information, communication with healthcare providers, media literacy, self-management, and decision-making, relate to proactive reproductive health behaviors. The findings are intended to guide future programming and policy that center cultural relevance, educational equity, and youth empowerment, particularly in hard-to-reach communities. By doing so, this research contributes to a growing global dialogue on achieving reproductive justice and health equity for all, especially among historically underserved and underrepresented populations.

## 2 Methods

### 2.1 Study design and participants

This cross-sectional study was conducted over 3 months, from January to March 2024, to examine health literacy and prevention behavior related to premature pregnancy among ethnic adolescent girls. The target population consisted of 156 ethnic female students enrolled in lower secondary schools (Grades 7–9). Based on Krejcie and Morgan's sample size determination (24), a minimum of 112 participants was required. To account for potential dropouts and incomplete responses, an additional 10% was added, resulting in a final size of 128 students. A proportionate stratified random sampling technique was employed to ensure proper representation of students from each school and grade level. Within each stratum, participants were then selected using simple random sampling until the desired number was reached. Inclusion criteria were as follows: participants had to be (1) female students of ethnic minority background; (2) currently studying in grades 7–9; (3) aged between years12 and 21 years to reflect late school entry or grade repetition common among ethnic minority adolescents; (4) able to listen, speak, read, and understand Thai; and (5) willing to voluntarily participate in the study. Exclusion criteria included students (1) diagnosed with cognitive or neurological disorders such as epilepsy, intellectual disability, developmental delay (2) with sensory impairments as confirmed by a medical professional, or (3) who were currently undergoing psychiatric treatment or had a history of psychiatric illness requiring treatment.

### 2.2 Research instruments

For data collection, we used a structured questionnaire based on a validated health literacy questionnaire for Thai females aged 15–21 (25). To more appropriately reflect the social and cultural realities of the ethnic groups in Northern Thailand, the instrument was modified. The final instrument consisted of 71 items across eight domains: General information, functional knowledge, access to health information and services, communication with health professionals, self-management of health conditions, media and information literacy, decision-making and appropriate action, pregnancy prevention behavior. Three subject matter experts reviewed the instrument for content validity. IOC values ranged from 0.67 to 1.00, with an average IOC of 0.67, indicating acceptable content validity. The instrument was pilot tested with 30 students with similar characteristics to the target group. Cronbach's alpha was 0.874, indicating high reliability and internal consistency.

### 2.3 Data analysis

Data was analyzed using descriptive and inferential statistical methods. Descriptive statistics were employed to summarize demographic characteristics, levels of health literacy, and pregnancy prevention behaviors. Categorical variables were presented using frequencies and percentages, whereas continuous variables were described using means and standard deviations. Before conducting inferential analyses, the assumptions of multiple linear regression were assessed to ensure the validity of the model. Normality of residuals was examined using histograms, Q–Q plots, and the Kolmogorov–Smirnov test. Linearity and homoscedasticity were evaluated using scatterplots of standardized residuals against predicted values. Multicollinearity was assessed using variance inflation factors (VIF), with all VIF values remaining below 2.0, indicating no significant multicollinearity concerns. These diagnostic checks confirmed that the regression model met the required assumptions. Pearson's correlation coefficient was used to explore bivariate relationships between health literacy domains and pregnancy prevention behavior. Multiple linear regression analysis was performed using the seven domains of health literacy—functional knowledge, access to health information and services, communication with health professionals, self-management of health conditions, media and information literacy, decision-making and appropriate action, and pregnancy prevention behavior as independent variables. Statistical significance was set at *p* < 0.05.

## 3 Result

The demographic characteristics, risky behaviors, and poor experiences of 128 ethnic students are shown in Table 1. The average age of the participants was 13.95 years (SD = 0.91). The Akha were the largest ethnic group (30.5%), followed by the Karen (24.2%), Lahu (17.2%), Yao (17.2%), and others (10.9%), which included the Shan, Wa, and Hmong. In terms of parental education, only 5.5% of students had parents with higher education, while most parents had graduated from primary school (28.9%) and lower secondary school (28.1%). Most parents worked in agriculture (47.7%) and labor (46.1%). Most students (62.5%) lived with their parents, followed by grandparents (17.2%) and other family members (20.3%). In terms of financial status, 11% reported different levels of financial inadequacy, while 57.8% reported having enough income with savings. 50.8% reported unsatisfactory life satisfaction, and nearly half (45.4%) had fair to inadequate academic performance. Regarding risk behaviors, 37.5% acknowledged skipping school, 31.3% reported frequently gaming, and 35.9% were drinking alcohol. 10.9% also reported having close friends who were involved in risky sexual activities. Remarkably, 60.2% of the participants said they had a family member who became pregnant before the age of 21, 4.7% said they had been forced into sex, and 24.2% said they had been the victim of verbal or physical sexual harassment. Details are shown in Table 1.

**Table 1:** Demographic characteristics, risk behaviors, and adverse experiences among ethnic female students (*N* = 128).

**Characteristics**	**No. (%)**
**Age Mean (SD)** = **13.95 (0.91)**	
**Ethnicity**	
Akha	39 (30.5%)
Karen	31 (24.2%)
Lahu	22 (17.2%)
Yao	22 (17.2%)
Others (Hmong, Shan, Wa)	14 (10.9%)
**Parental education**	
No formal education	31 (24.2%)
Primary education	37 (28.9%)
Lower secondary	36 (28.1%)
Secondary/vocational upper	17 (13.3%)
Higher education	7 (5.5%)
**Parental occupation**	
Agriculture	61 (47.7%)
Labor	59 (46.1%)
Own business	8 (6.3%)
**Living with**	
Parents (father/mother)	80 (62.5%)
Grandparents	22 (17.2%)
Other relatives (aunt, uncle, sibling)	26 (20.3%)
**Family economic status**	
Adequate with savings	74 (57.8%)
Adequate, no savings	40 (31.3%)
Inadequate with some debt	9 (7.0%)
Inadequate with major debt	5 (3.9%)
Academic performance (fair to poor)	58 (45.4%)
Life satisfaction: neutral or unsatisfied	65 (50.8%)
**Risk behaviors**	
Ever consumed alcohol	46 (35.9%)
Addicted to games (sometimes/often)	40 (31.3%)
Skipping school (sometimes/often)	48 (37.5%)
Close to peers with risky sexual behavior	14 (10.9%)
**Risk behaviors**	
Ever experienced verbal or physical sexual harassment	31 (24.2%)
Ever coerced into sex	6 (4.7%)
Have someone in the family who is pregnant before age 21	77 (60.2%)

Table 2 shows the descriptive statistics for each component of health literacy and pregnancy prevention among ethnic students. The results indicate that students reveal levels of partly correct functional knowledge (mean = 5.13). However, it was found that many of the domains were lacking, such as access to health information, communication with health professionals, self-management of medical conditions, and media and information literacy. However, students excelled in the areas of decision-making and appropriate action (mean = 31.44) and pregnancy prevention behavior (mean = 68.80), both of which were evaluated as excellent. These findings highlight the necessity of enhancing foundational and active literacy skills to promote health behaviors more effectively.

**Table 2:** Descriptive statistics of health literacy components and pregnancy prevention behavior (*N* = 128).

**Variable**	**Min**	**Max**	**Mean**	**SD**	**Level**
Functional knowledge	0.00	8.00	5.13	1.63	Partly correct
Access to health information and services	7.00	21.00	12.80	3.51	Inadequate
Communication with health professionals	7.00	27.00	16.19	2.83	Inadequate
Self-management of health conditions	5.00	25.00	11.82	5.58	Inadequate
Media and information literacy	5.00	25.00	10.35	5.18	Inadequate
Decision-making and appropriate action	17.00	36.00	31.44	4.16	Excellent
Pregnancy prevention behavior	36.00	74.00	68.80	4.69	Excellent

The three components of students' health literacy, categorized as cognitive (basic), interactive (social), and critical (judgmental), were found to differ significantly. Moreover, 63.3% of students performed poorly in the cognitive domain and 71.9% in the interactive domain; a significant percentage of students showed inadequate health literacy. This outcome suggests difficulties in obtaining, understanding, and communicating health information effectively. Conversely, performance in the critical domain was relatively robust; although merely 14.8% reached a high level, a significant majority (66.4%) achieved moderate proficiency in assessing and utilizing health information. These results point to the importance of targeted interventions to enhance both the fundamental and social aspects of health literacy, particularly in helping adolescents make informed reproductive health choices with increased confidence and autonomy (Table 3).

**Table 3:** Student distribution by health literacy levels in cognitive, interactive, and critical domains (*N* = 128).

**Health literacy domain**	**Basic (cognitive) no. (%)**	**Interactive (social) no. (%)**	**Critical (judgmental) no. (%)**
Poor (not sufficient)	81 (63.3%)	92 (71.9%)	24 (18.8%)
Fair (moderate)	45 (35.2%)	30 (23.4%)	85 (66.4%)
Good (high)	2 (1.6%)	6 (4.7%)	19 (14.8%)

Table 4 shows the correlation analysis that revealed significant relationships among several components of health literacy and pregnancy prevention behavior. Notably, decision-making skills showed a moderate positive correlation with pregnancy prevention behavior (*r* = 0.336, *p* < 0.01), indicating that students with stronger decision-making abilities were more likely to engage in practical preventive behavior. Functional knowledge shows weak and insignificant correlations with most other aspects, as well as with prevention behavior (*r* = 0.065), suggesting that merely possessing basic knowledge may not directly influence people's actions. Additionally, a strong correlation was found between access to health information, consulting health professionals, self-management, and media literacy (*r* values ranging from 0.548 to 0.755, *p* < 0.001), indicating that these advanced health skills are interrelated. However, some areas, such as consulting health professionals and self-managing health, had significant negative relationships with prevention behavior, which might indicate that some deeper issues or challenges make it difficult to apply these skills in real life. These findings highlight how important it is for young people to make good decisions about pregnancy prevention and suggest that we need to improve practical skills beyond just basic knowledge so that teenagers can make informed and suitable choices about their reproductive health.

**Table 4:** Pearson's correlation matrix among health literacy components and pregnancy prevention behavior (*N* = 128).

**Component**	**Functional knowledge**	**Access to info and services**	**Communication with health professionals**	**Self-management**	**Media literacy**	**Decision-making**	**Prevention behavior**
Functional knowledge	1	0.058	0.024	0.189^*^	0.181^*^	0.298^**^	0.065
Access to info and services		1	0.625^**^	0.691^**^	0.551^**^	0.177^*^	−0.130
Communication with health professionals			1	0.616^**^	0.548^**^	0.073	−0.247^**^
Self-management				1	0.755^**^	0.177^*^	−0.205^*^
Media literacy					1	0.125	−0.124
Decision-making						1	0.336^**^
Prevention behavior							1

* *p* < 0.05;

** *p* < 0.001.

**Figure 1:**
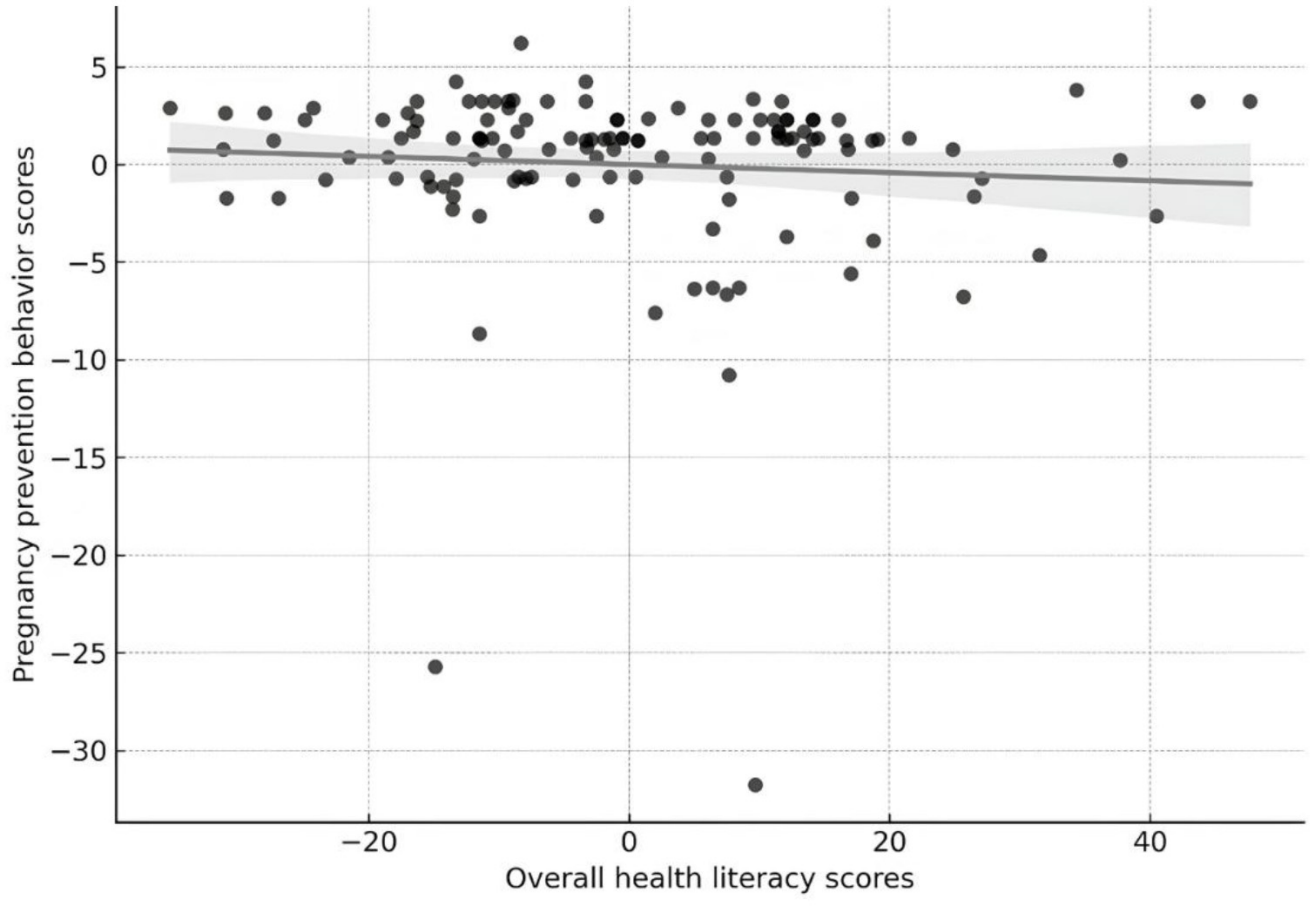
The partial correlation between overall health literacy and pregnancy prevention behavior (controlling for age and education).

Figure 1 shows the partial relationship between ethnic students' overall health literacy scores and their scores on pregnancy prevention behavior, after adjusting for age and education. The scatterplot shows the remaining values from linear regression models. The analysis displayed an inadequate and non-significant negative partial correlation (*r* = −0.074, *p* = 0.408) between the two variables after adjusting for the covariates.

As shown in Table 5, the multiple linear regression analysis examined the extent to which the three dimensions of health literacy are basic, interactive, and critical predicted pregnancy prevention behavior among ethnic minority adolescent girls. The overall model was statistically significant, *F* (3, 101) = 8.973, *p* < 0.001, accounting for 17.8% of the variance in pregnancy prevention behavior (*R*^2^ = 0.178). Among the predictors, critical health literacy was a significant positive predictor of pregnancy prevention behavior (*B* = 0.292, β = 0.438, *p* < 0.001), indicating that students with higher levels of evaluative and judgment-based literacy were more likely to engage in effective prevention behaviors. In contrast, interactive health literacy showed a significant negative association with pregnancy prevention behavior (*B* = −0.348, β = −0.568, *p* < 0.001). This finding suggests that interpersonal communication and help-seeking in this context may not translate into improved preventive behaviors, possibly reflecting community-level misinformation, cultural taboos, or inconsistent reproductive health messaging. Basic health literacy was not a significant predictor of pregnancy prevention behavior (*B* = 0.073, β = 0.061, *p* = 0.606), implying that foundational knowledge alone did not meaningfully influence prevention behaviors. Overall, these findings indicate that different dimensions of health literacy exert distinct and sometimes opposing influences on adolescent pregnancy prevention behaviors. Strengthening students' critical health literacy appears particularly important for fostering effective reproductive decision-making, while interventions aimed at improving interactive literacy may require culturally sensitive redesign to address barriers embedded in social communication processes.

**Table 5:** The results of a multiple linear regression analysis examining the association between dimensions of health literacy and pregnancy prevention behavior.

**Predictor**	***B***	**Std error**	**β**	***t***	***p*-Value**
Basic health literacy	0.0729	0.141	0.061	0.517	0.606
Interactive health literacy	−0.3482	0.078	−0.568	−4.473	<0.001
Critical health literacy	0.292	0.073	0.438	4.005	<0.001

Model summary: R^2^ = 0.178, F (3, 101) = 8.973, *p* < 0.001.

## 4 Discussion

Our study revealed a compelling paradox: while ethnic students in Northern Thailand demonstrated limited basic and interactive health literacy, they exhibited strong decision-making skills and high levels of pregnancy prevention behavior. This finding is central to understanding how health knowledge, in its various dimensions, influences the pregnancy prevention behaviors of female ethnic students in this specific context. Key elements of this dynamic include the robust relationship observed between decision-making ability and pregnancy prevention behavior, along with the influence of limited foundational health knowledge, risky behaviors, and systemic barriers affecting access to reproductive health services. Although the regression model significantly predicted pregnancy prevention behavior, the *R*^2^ value was relatively low, indicating that other unmeasured factors or potential nonlinear relationships may also influence adolescent's prevention behaviors.

### 4.1 The disparity between health literacy and preventive behaviors

Despite overall low levels of health literacy, particularly in domains such as access to health information, communication with health professionals, self-management of health conditions, and media and information literacy, students showed notably strong scores in decision-making and pregnancy prevention behavior. Previous studies have shown that adolescent pregnancy is associated with increased risks of low birth weight, perinatal mortality, anemia, and long-term socioeconomic disadvantage (5–7). These outcomes were consistent with the patterns observed in the ethnic adolescent population in this study. This observed difference suggests that practical health behaviors can manifest even without comprehensive foundational health knowledge, especially within groups where cultural backgrounds play a significant role (26). The conceptualize health literacy as a multidimensional construct, where individuals may excel in one area while lacking proficiency in another (27). In the unique context of ethnic communities, decision-making skills related to health may be culturally guided, potentially through established family norms or peer networks, which could compensate for gaps in formal education or digital health literacy (21). However, relying solely on these culturally guided behaviors might not be sustainable if critical and functional health-related skills are not simultaneously strengthened, particularly when adolescents encounter complex or unfamiliar health situations (28). Therefore, tailored interventions are essential to promote analytical and critical thinking, empowering students to evaluate diverse information sources and engage in proactive, practical actions. The multiple linear regression findings further illuminated the distinct roles of various health literacy dimensions. While basic health literacy showed no significant association with pregnancy prevention behavior, critical health literacy emerged as a positive and significant predictor (β = 0.438, *p* < 0.001). This aligns with previous research that emphasizes the importance of evaluative thinking in health-related decision-making (29). Unexpectedly, interactive health literacy demonstrated a significant negative association (β = −0.568, *p* < 0.001) with pregnancy prevention behavior. The negative association between interactive literacy and prevention behavior may reflect culturally mediated communication barriers. Among ethnic adolescents, interpersonal discussions around sexuality may transmit misinformation or reinforce restrictive norms, which could explain why higher levels of interactive engagement did not correspond to better preventive behaviors. This counterintuitive finding suggests that interpersonal communication within these communities, which is a core aspect of interactive literacy, may inadvertently transmit conflicting, non-evidence-based, or even stigmatizing messages regarding reproductive health. This underscores a critical need to not only strengthen critical thinking in culturally nuanced ways but also to reframe peer and family communication within future reproductive health interventions, potentially through structured school-based discussions or peer education programs.

### 4.2 Socio-cultural and structural risk factors that influence adolescent reproductive behavior

Our study results also highlighted significant socio-cultural and structural risk factors. A substantial proportion of students (60.2%) reported a family history of teenage pregnancies, and nearly half (45.4%) demonstrated fair academic performance. These findings are consistent with studies on hill tribe adolescents by Tamornpark et al., (30) which identified intergenerational risk patterns as a key factor influencing adolescent reproductive behavior. which identified intergenerational risk patterns as a key factor influencing adolescent reproductive behavior. Furthermore, only a small percentage (5.5%) of the participants' parents had attained a higher level of education. Research consistently demonstrates a strong relationship between parental education and adolescent health behaviors, which in turn profoundly influences the literacy environment and health communication within families (31). Previous study have shown that targeted institutional interventions can help offset familial disadvantages in early pregnancy prevention (32). Another compelling result was the prevalence of various risk behaviors and adverse experiences among the participants: 37.5% acknowledged skipping school, 35.9% reported alcohol consumption, and 24.2% disclosed having experienced verbal or physical sexual harassment. These findings collectively highlight significant environmental vulnerabilities and underscore how prevalent socio-cultural stigma, and a lack of supportive structures can hinder access to effective sexual and reproductive health services (33). The occurrence of coerced sex (4.7%) further emphasizes the urgent need for comprehensive protective measures and support systems.

### 4.3 Strategic directions for promoting reproductive health

The observed pattern of strong decision-making skills alongside low basic and interactive health literacy challenges traditional public health frameworks that view comprehensive health knowledge as the sole foundation for all health behaviors (34). However, studies conducted in Thailand and Laos have suggested that deeply ingrained cultural norms and community expectations can, in certain contexts, serve as alternative drivers for health behaviors, sometimes compensating for a lack of formal health knowledge (35, 36). When considered together, the findings of this study strongly support the view that promoting reproductive health among ethnic adolescents requires approaches that extend beyond conventional knowledge-based models. Effective programs and models must holistically address cultural barriers, specific behavioral risks, emotional readiness, and all dimensions of health literacy (37). This aligns with global trends and challenges faced by ethnic minority youth, who often encounter complex access barriers such as provider bias, cultural taboos, and geographic isolation, as highlighted by the World Health Organization (WHO) (38). Recent evidence highlights the importance of culturally sensitive communication within healthcare systems. Brooks et al. (39) emphasize that culturally sensitive communication involves tailored messaging, meaningful community engagement, and adaptation of health information to local cultural contexts. The emphasize the crucial role of effective communication between healthcare providers and patients, particularly within underserved communities (40). Furthermore, the education system has a vital role in reducing health inequalities, as the national curriculum can help mitigate disparities in adolescent health outcomes (41). Consistent with our findings, Okan et al. (26) proposed a comprehensive model that integrates personal, social, and systemic aspects of health literacy to improve youth decision-making.

### 4.4 Implications for global health policy and practice

The findings of this study have significant implications that extend beyond the local context of Northern Thailand. Ethnic minority adolescents in this region share similar barriers to reproductive health access as marginalized youth in other low- and middle-income countries (LMICs), including limited access to culturally relevant health information, language barriers, geographic isolation, and deeply embedded social norms. As such, these insights align closely with the global priorities set by the World Health Organization (WHO) and the United Nations Population Fund (UNFPA), which emphasize the critical importance of tailoring reproductive health interventions to the unique cultural and contextual realities of vulnerable populations (42, 43). The observed gap between basic health knowledge and actual prevention behavior powerfully underscores the need for policies that transcend conventional, didactic education models. Instead of focusing solely on content delivery, health education strategies should prioritize strengthening decision-making skills, critical thinking, and media literacy—components that showed stronger associations with positive behavioral outcomes in this study. This finding is consistent with prior research, which suggests that commonly accepted sexual and reproductive health (SRH) interventions may be ineffective if they neglect context-specific and skill-based approaches (44). Moreover, the strong correlation between decision-making ability and pregnancy prevention behavior highlights the urgency of actively including adolescent voices and perspectives in the design and implementation of reproductive health programs. Culturally sensitive, youth-centered strategies, delivered through peer-led models, mobile health services, and community-based initiatives, hold immense potential to bridge the existing gap between mainstream health systems and underserved adolescents (45).

Finally, our results reinforce the global call for multisectoral collaboration. Public health authorities, education ministries, and local community leaders must work in concert to ensure that reproductive health interventions are not only accessible but also culturally relevant, sensitive, and meaningful to the lives of ethnic adolescents. Integrating essential life skills into national curricula and applying communication frameworks recommended by global bodies, such as UNESCO and WHO, will be fundamental to achieving Sustainable Development Goal 5.6 and improving sexual and reproductive health outcomes for vulnerable youth worldwide (41, 46). Although the regression model significantly predicted pregnancy prevention behavior, the *R*^2^ value was relatively low, suggesting that additional unmeasured constructs may contribute to these behaviors. Future studies could apply PLS-SEM to validate and extend the structural pathways suggested by the current findings, using the conceptual relationships identified in this study as a foundation for more complex modeling.

## 5 Strengths and limitations of the study

This study provides valuable insights into health literacy and pregnancy prevention among ethnic students in Northern Thailand, a vulnerable population. However, being a cross-sectional study, it cannot establish causality. Limitations also include reliance on self-reported data and restricted generalizability due to the focus on in-school adolescents from a single province. Future research could benefit from longitudinal or mixed methods approaches. Beyond structural modeling approaches such as PLS-SEM, future research may incorporate clustering or machine-learning classification approaches to identify high-risk subgroups based on demographic, familial, or behavioral patterns. Such analytic techniques could support government agencies in prioritizing targeted interventions for specific adolescent profiles. Moreover, given the relatively low *R*^2^ value observed in this study, non-linear modeling approaches such as logistic regression or generalized additive models may provide a more nuanced understanding of complex relationships that linear models cannot fully explain.

## 6 Recommendations for practice and policy

Given the moderate explanatory power of critical health literacy, future interventions should not only focus on information provision but also incorporate critical thinking modules into school curricula, particularly through peer-led participatory workshops.

## 7 Conclusion

This study underscores the importance of enhancing decision-making and critical health literacy to improve pregnancy prevention behaviors among ethnic adolescents. While basic knowledge remains limited, culturally grounded and skill-based interventions may be more effective in fostering behavior change. Tailored strategies that consider sociocultural contexts are crucial for reducing disparities and promoting reproductive health equity. These findings support global efforts to achieve Sustainable Development Goal 5.6, which calls for universal access to sexual and reproductive health services and aligns with WHO's call for context-specific health literacy promotion among vulnerable populations.

## Data Availability

The original contributions presented in the study are included in the article/supplementary material, further inquiries can be directed to the corresponding author.

## References

[1] Daniels S Robson D Flatley C Kumar S. Demographic characteristics and pregnancy outcomes in adolescents - experience from an Australian perinatal center. Aust N Z J Obstet Gynaecol. (2017) 57:630–5. doi: 10.1111/ajo.12651 28635013

[2] World Health Organization. Adolescent Pregnancy. (2023). Available online at: https://rh.anamai.moph.go.th/ (Accessed October 12, 2024).

[3] Plesons M Bastien S Dyalchand A Mehta R Speizer IS Chandra-Mouli V. Updated world health organization guideline on preventing early pregnancy and poor reproductive outcomes among adolescents in low- and middle-income countries. J Adolesc Health. (2025) 77:803–9. doi: 10.1016/j.jadohealth.2025.07.024 40956266 PMC12586972

[4] World Health Organization. The Adolescent Health Indicators Recommended by the Global Action for Measurement of Adolescent Health: Guidance for Monitoring Adolescent Health at Country, Regional and Global Levels. Geneva: World Health Organization (2024).

[5] World Health Organization. World Health Statistics 2019: Monitoring Health for the SDGs, Sustainable Development Goals. (2019). Available online at: https://digitallibrary.un.org/record/3868814 (Accessed November 4, 2024).

[6] Wong SP Twynstra J Gilliland JA Cook JL Seabrook JA. Risk factors and birth outcomes associated with teenage pregnancy: a Canadian sample. J Pediatr Adolesc Gynecol. (2020) 33:153–9. doi: 10.1016/j.jpag.2019.10.006 31634579

[7] Chakole S Akre S Sharma K Wasnik P Wanjari MB. Unwanted teenage pregnancy and its complications: a narrative review. Cureus. (2022) 14:e32662. doi: 10.7759/cureus.32662 36686124 PMC9848684

[8] United Nations Development Programme. Sustainable Development Goals. (2021). Available online at: https://www.undp.org/sustainabledevelopmentgoals/ (Accessed October 12, 2024).

[9] Reproductive Health Bureau. The 2^*nd*^ *National Reproductive Health Development Policy and Strategy (2017-2026) on the Promotion of Quality Birth and Growth*. (2026). Available online at: https://hhdc.anamai.moph.go.th/th/ (Accessed October 15, 2024).

[10] UNICEF. Review of Comprehensive Sexuality Education in Thailand. Bangkok: UNICEF Thailand Country Office (2016).

[11] Chiang Rai Provincial Cultural Office. Ethnic Groups in Chiang Rai Province. (2021). Available online at: https://online.anyflip.com/pwpje/oeeh/mobile/ (Accessed November 4, 2024).

[12] The Health Center of Ethnic Groups, Marginal People, and Migrant Workers. Annual Health Report on Ethnic Groups. (2020). Available online at: https://childsdream.org/wp-content/uploads/2015/03/LWO-Lahu-Womens-Organisation_Health_Fact-Sheet_0219.pdf (Accessed October 15, 2024).

[13] Lahu Women Organization. Health Awareness Programme. (2015). Available online at: https://hed.hss.moph.go.th/ (Accessed October 15, 2024).

[14] Princess Maha Chakri Siridhorn Anthropology Center. Hill Tribe. (2024). Available online at: http://www.sac.or.th/main/index.php (Accessed November 23, 2024).

[15] Banati P Jones N Moreau C Mmari K Kågesten A Austrian K . Intersectionality, gender norms, and young adolescents in context: a review of longitudinal multicountry research programmes to shape future action. Lancet Child Adolesc Health. (2024) 8:522–31. doi: 10.1016/S2352-4642(24)00079-8 38897717

[16] UNICEF. Situation Analysis of Adolescent Pregnancy in Thailand: Synthesis Report 2015. Bangkok: UNICEF Thailand (2015).

[17] Nutbeam D Lloyd JE. Understanding and responding to health literacy as a social determinant of health. Annu Rev Public Health. (2021) 42:159–73. doi: 10.1146/annurev-publhealth-090419-102529 33035427

[18] Liu J Zhao S Chen X Falk E Albarracín D. The influence of peer behavior as a function of social and cultural closeness: a meta-analysis of normative influence on adolescent smoking initiation and continuation. Psychol Bull. (2017) 143:1082–115. doi: 10.1037/bul0000113 28771020 PMC5789806

[19] McKenna VB Sixsmith J Barry MM. The relevance of context in understanding health literacy skills: findings from a qualitative study. Health Expect. (2017) 20:1049–60. doi: 10.1111/hex.12547 28402013 PMC5600250

[20] Dongarwar D Salihu HM. Influence of sexual and reproductive health literacy on single and recurrent adolescent pregnancy in Latin America. J Pediatr Adolesc Gynecol. (2019) 32:506–13. doi: 10.1016/j.jpag.2019.06.003 31195100

[21] Alhussaini NW Elshaikh U Abdulrashid K Elashie S Hamad NA Al-Jayyousi GF. Sexual and reproductive health literacy of higher education students: a scoping review of determinants, screening tools, and effective interventions. Global Health Action. (2025) 18. doi: 10.1080/16549716.2025.2480417 40116037 PMC11934179

[22] Jungpichanvanich S. Situation and measures to solve the problems relating to teenage pregnancy in Mae Suai District, Chiang Rai Province. J Health Sci. (2014) 23:643.

[23] Suratana S Boonchiang W Apidechkul T Naksen W Mulikaburt T Chomsri P . Community-based reproductive health care model effectively enhances reproductive health among Lahu women in Northern Thailand. J Racial Ethn Health Disparities. (2024). doi: 10.1007/s40615-024-01959-5 Available online at: https://link.springer.com/article/10.1007/s40615-024-01959-5 PMC11913957 38421508

[24] Krejcie RV Morgan DW. Determining sample size for research activities. Educ Psychol Meas. (1970) 30:607–10. doi: 10.1177/001316447003000308

[25] Health Education Division, Department of Health Service Support, Ministry of Public Health. Assess Health Literacy to Prevent Premature Pregnancy for Thai Adolescent Women Aged 15-21 Years. (2014). Available online at: https://iris.who.int/handle/10665/44344 (Accessed November 4, 2024).

[26] Okan O Bauer U Levin-Zamir D Pinheiro P Sørensen K. International Handbook of Health Literacy: Research, Practice and Policy Across the Lifespan. Policy Press (2019). p. 764. doi: 10.56687/9781447344520

[27] Kour S Jyoti J. Cross-cultural training and adjustment through the lens of cultural intelligence and type of expatriates. Employee Relations. (2022) 44:1–36. doi: 10.1108/ER-07-2020-0355

[28] Fleary SA Joseph P. Adolescents' health literacy and decision-making: a qualitative study. Am J Health Behav. (2020) 44:392–408. doi: 10.5993/AJHB.44.4.3 32553022 PMC8191815

[29] Wani SA Hussian Z. Developing Critical Thinking Skills: Encouraging Analytical and Creative Thinking. Hershey, PA: IGI Global (2024). p. 114–30. doi: 10.4018/979-8-3693-0868-4.ch007

[30] Tamornpark R Apidechkul T Upala P Chomchoei C Yeemard F. Factors influencing early sexual initiation among hill tribe youths in Chiang Rai Province, Northern Thailand: a community-based cross-sectional study. PLoS ONE. (2025) 20. doi: 10.1371/journal.pone.0321083 40198628 PMC11991288

[31] Csima M Podráczky J Keresztes V Soós E Fináncz J. The role of parental health literacy in establishing health-promoting habits in early childhood. Children. (2024) 11:576. doi: 10.3390/children11050576 38790571 PMC11119361

[32] Brindis CD Decker MJ Gutmann-Gonzalez A Berglas NF. Perspectives on adolescent pregnancy prevention strategies in the United States: looking back, looking forward. Adolesc Health Med Ther. (2020) 11:135–45. doi: 10.2147/AHMT.S219949 33117030 PMC7567553

[33] Kinaro JW Wangalwa G Karanja S Adika B Lengewa C Masitsa P. Socio-cultural barriers influencing utilization of sexual and reproductive health (SRH) information and services among adolescents and youth 10-24 years in pastoral communities in Kenya. Adv Sex Med. (2019) 9:1–16. doi: 10.4236/asm.2019.91001

[34] Wiwatkamonchai A Mesukko J Klunklin P Fongkaew W. Youths' perceptions regarding access to sexual and reproductive health services. Pac Rim Int J Nurs Res Thail. (2023) 27:121–37. doi: 10.60099/prijnr.2023.260337

[35] Narkbubpha R Sriyasak A Sarakshetrin A Thungthin P. Assessing sexual health literacy among Thai female adolescents in non-formal education: a mixed-methods study. Belitung Nurs J. (2025) 11:340–8. doi: 10.33546/bnj.3744 40438661 PMC12107267

[36] Vongxay V Albers F Thongmixay S Thongsombath M Broerse JEW Sychareun V . Sexual and reproductive health literacy of school adolescents in Lao PDR. PLoS ONE. (2019) 14:e0209675. doi: 10.1371/journal.pone.0209675 30650100 PMC6334956

[37] Awoonor-Williams JK Vaughan-Smith MN Phillips JF. Social Determinants of Sexual and Reproductive Health: Informing Future Research and Programme Implementation. Geneva: World Health Organization (2010). Available online at: https://iris.who.int/handle/10665/44344

[38] World Health Organization. Social Determinants of Sexual and Reproductive Health. Informing Future Research and Programme Implementation. Geneva, Switzerland: WHO (2010). Available online at: http://www.ncdsv.org/images/WHO_SocialDeterminantsSexualHealth_2010.pdf (Accessed November 14, 2024).

[39] Brooks LA Manias E Bloomer MJ. Culturally sensitive communication in healthcare: a concept analysis. Collegian. (2019) 26:383–91. doi: 10.1016/j.colegn.2018.09.007

[40] Sim D Yuan SE Yun JH. Health literacy and physician-patient communication: a review of the literature. Int J Commun Health. (2016) 10:101–14.

[41] World Health Organization. Operational Framework for Primary Health Care: Transforming Vision into Action. Geneva: World Health Organization (2020).

[42] Kollodge R editor. My Body is My Own: Claiming the Right to Autonomy and Self-Determination. New York, NY: United Nations Population Fund (2021).

[43] Chandra-Mouli V Lane C Wong S. What does not work in adolescent sexual and reproductive health: a review of evidence on interventions commonly accepted as best practices. Glob Health Sci Pract. (2015) 3:333–40. doi: 10.9745/GHSP-D-15-00126 26374795 PMC4570008

[44] Patton GC Sawyer SM Santelli JS Ross DA Afifi R Allen NB . Our future: a Lancet commission on adolescent health and wellbeing. Lancet. (2016) 387:2423–78. doi: 10.1016/S0140-6736(16)00579-1 27174304 PMC5832967

[45] United Nations Educational, Scientific and Cultural Organization. International Technical Guidance on Sexuality Education: An Evidence-Informed Approach. Paris: UNESCO (2018).

[46] National Academies of Sciences, Engineering, Medicine, Health, Medicine Division, Division of Behavioral, et al. The Promise of Adolescence: Realizing Opportunity for All Youth. Backes EP ., editors. London: National Academies Press (US) (2019). 31449373

